# Social context shapes acoustic signalling in the territorial damselfish *Stegastes fuscus*

**DOI:** 10.1111/jfb.70651

**Published:** 2026-09-14

**Authors:** Aléxia A. Lessa, Viviane Barroso, Fábio C. Xavier, Carlos E. L. Ferreira, Eric Parmentier

**Affiliations:** ^1^ Programa de Pós‐Graduação em Ecologia Universidade Federal do Rio de Janeiro Rio de Janeiro Brazil; ^2^ Reef Systems Ecology and Conservation Lab, Department of Marine Biology Federal Fluminense University Niterói Brazil; ^3^ Laboratory of Functional and Evolutionary Morphology, Department of Biology, Ecology, Evolution University of Liège Liège Belgium

**Keywords:** acoustic communication, Pomacentridae, reef fish, territorial behaviour, vocal repertoire

## Abstract

Acoustic communication plays an important role in social interactions in reef fishes, but how signals vary with behavioural context remains poorly understood for many species. We characterised the acoustic repertoire of the Brazilian damselfish *Stegastes fuscus* using synchronised underwater audio–video recordings and tested how sound features vary across social behaviours. Sounds were recorded during agonistic and courtship‐related interactions, and temporal and spectral acoustic parameters were measured to compare acoustic structure among contexts. Differences between behaviours were primarily found in temporal features: Courtship sounds were longer and contained more pulses than agonistic sounds. These results show that *S. fuscus* conveys social context mainly through modulation of temporal organisation rather than spectral properties. Our findings indicate that a single call type can be flexibly adjusted to signal behavioural context during territorial defence and courtship. By documenting the acoustic repertoire of a dominant territorial herbivore, this study highlights how social context structures sound production in damselfishes.

## 1 INTRODUCTION

Acoustic communication is widespread among teleost fishes and plays an important role in social interactions such as mate attraction and territorial defence (Amorim et al., 2006; Hastings & Thomas, 2025; Ladich, 2014). Variation in acoustic signals across behavioural contexts is central to animal communication, allowing individuals to signal motivational state, reproductive condition, or aggressive intent (Looby et al., 2023). In reef environments, many species emit short pulsed sounds during close‐range agonistic and reproductive behaviours (Boyle & Cox, 2009; Tricas & Boyle, 2014), where acoustic signals complement visual displays (Ruppé et al., 2015). This suggests that communication in reef fishes often relies on multimodal signalling systems.

Damselfishes (Pomacentridae) stand out as one of the best‐studied reef fish families in terms of acoustic communication. Many species produce conspicuous pulsed sounds that are closely associated with clearly identifiable behaviours and multimodal displays. These often include transient colour changes, making such species ideal models for linking acoustic signals to social context (Lobel et al., 2010; Parmentier et al., 2016). In damselfishes, aggressive and courtship sounds are generated by rapid jaw closure driven by the cranio‐mandibular dorsal (c‐md) ligament, which connects the hyoid apparatus to the mandible. During simultaneous neurocranial elevation and hyoid depression, the c‐md ligament acts as a tensioned cord that forces abrupt mouth closure. The collision of the upper and lower jaw teeth produces short, pulsed sounds (Colleye & Parmentier, 2012; Olivier et al., 2014, 2015; Parmentier et al., 2007). Both males and females commonly emit sounds during agonistic interactions related to territory defence (Colleye & Parmentier, 2012; Myrberg, 1972), whereas sound production during reproductive contexts is often male‐biased (Lobel & Mann, 1995). Across species, these signals typically consist of short, pulsed sounds whose temporal structure varies according to behavioural context. In particular, courtship sounds are often composed of more pulses than agonistic sounds, reflecting differences in signalling function between social contexts (Mann & Lobel, 1998; Parmentier et al., 2010).

Although acoustic behaviour has been described in several genera of this family, including *Dascyllus* (Parmentier et al., 2009, 2010), *Pomacentrus* (Myrberg, 1997; Myrberg et al., 1986) and *Amphiprion (*Colleye & Parmentier, 2012; Laboury et al., 2023; Parmentier et al., 2005, 2007), detailed quantitative analyses remain limited for many species of *Stegastes* (Parmentier et al., 2016). Early studies reported pulsed courtship sounds with broadly overlapping characteristics among sympatric species (Myrberg, 1972; Myrberg & Spires, 1972; Spanier, 1979), generally within a frequency range of approximately 250–1500 Hz. Auditory studies further indicated peak sensitivity around 500 Hz (Egner & Mann, 2005; Myrberg & Spires, 1980). However, the structure, diversity, and contextual use of vocal signals remain insufficiently documented in most *Stegastes* species. Because acoustic signals are often species‐specific and context‐dependent, detailed behavioural and acoustic descriptions are necessary to fully characterise communication systems within the genus.

The Brazilian damselfish *Stegastes fuscus* (Cuvier 1830) is the most abundant territorial herbivore on shallow reefs along the Brazilian coast (Chaves et al., 2021). This species plays a key ecological role in structuring benthic communities through its intense grazing and territorial behaviour (Ferreira et al., 1998). By aggressively defending their territory and excluding other herbivorous fishes, individuals of *S. fuscus* promote the development of diverse algal assemblages and associated cryptofauna within their territories, while also enhancing local primary productivity (Ferreira et al., 1998). Given its ecological importance and frequent sound production, characterising the acoustic repertoire of *S*. *fuscus* is essential for understanding how acoustic communication mediates key behaviours in a functionally important reef fish. Such information is also important for future acoustic monitoring of reef systems, particularly in coastal environments increasingly affected by anthropogenic noise (Duarte et al., 2021).

Here, we use in situ synchronous audio–video recordings to characterise the acoustic repertoire of the endemic Brazilian damselfish *S. fuscus*, describing both the structure of its vocal signals and their behavioural context. Because agonistic and reproductive interactions differ in their social function, we tested whether acoustic signals vary across social contexts, predicting that calls produced during courtship would be longer and contain more pulses than agonistic calls.

## 2 MATERIALS AND METHODS

### 2.1 Ethics statement

Field observations were non‐invasive and complied with Brazilian regulations for wildlife research. Sampling was authorised by the Brazilian Institute of Environment and Natural Resources (SISBIO licence no. 88256‐1). Disturbance to animals was minimised by slowly approaching focal territories during equipment deployment and by leaving the area immediately after positioning the acoustic recorder and camera, allowing recordings to proceed without human presence. Equipment retrieval followed the same procedure.

No formal ethical approval was required for this study because it involved non‐invasive behavioural observations of wild fish without handling or manipulation. This was confirmed by institutional guidelines. All procedures followed relevant national legislation and guidelines for the use of animals in research.

### 2.2 Field recordings

Field recordings were made at three different subtropical rocky reefs of the Arraial do Cabo Marine Reserve, Brazil (23°44′S, 42° W) (Figure 1). Recording sessions lasted between 70 and 120 min and were conducted during daylight hours in January, February, and December 2025, at depths ranging from 3 to 5 m. Each session included recordings of multiple individuals for at least 15 min. Because individuals were not captured or marked, it was not possible to determine the exact number of calling individuals. However, recordings were obtained from multiple territories across different sites and sessions, thereby increasing the representation of different individuals in the dataset. Recordings were conducted by a snorkeller who approached focal territories slowly to minimise disturbance and deployed both the Hydromoth underwater recorder (sampling rate 48 kHz, medium‐gain, 16 bits‐sample) and a GoPro Hero 10 camera (1080p, 30 frames s^−1^) at approximately 1 m from the focal area. After deployment, the snorkeller left the area, allowing recordings to proceed without human presence. All recordings were conducted in shallow reef areas accessible from shore, without the use of boats. Simultaneous video recordings were made using GOPRO Hero 10 cameras placed at a similar distance. After deploying the recording devices, the observers left the area to avoid any external disturbances altering the fish's behaviour.

**FIGURE 1:**
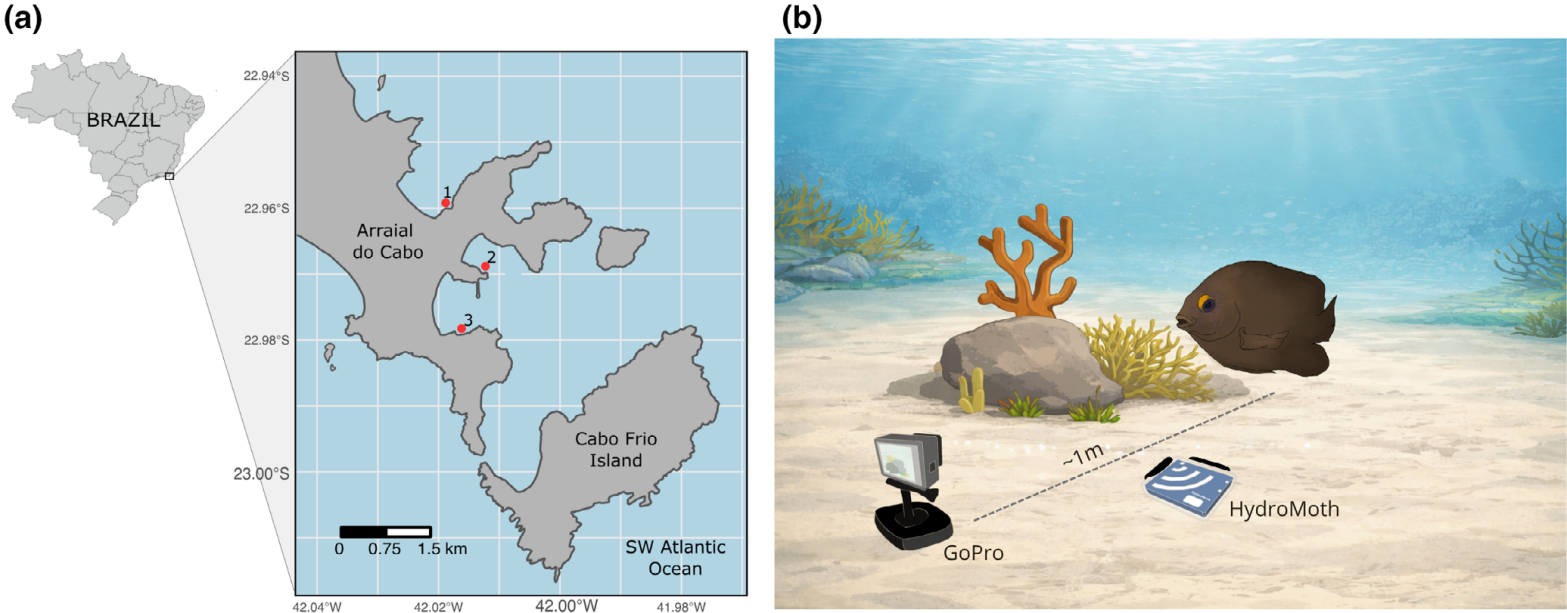
Study area and underwater recording set‐up used for behavioural and acoustic observations of *Stegastes fuscus*. (a) Map of the study area showing the recording sites in red: (1) Prainha, (2) Forno, and (3) Anjos. (b) Schematic illustration of the underwater set‐up used for synchronised audio–video recordings in the field.

### 2.3 Video and audio analysis

A total of 671 min of video recordings obtained from 39 recording sessions were analysed (Table 1). The video analysis was conducted by independently observing the video recordings to catalogue and characterise behavioural events associated with sound production. Soundtracks were extracted in .wav format from the videos analysed in DaVinci Resolve and sounds were isolated from these soundtracks. The audio and video recordings were synchronised during post‐processing in DaVinci Resolve using automatic waveform‐based audio synchronisation between the GoPro audio track and the Hydromoth recording. Only calls recorded by the Hydromoth underwater recorder with a high signal‐to‐noise ratio were included in the analysis. Consequently, the final acoustic dataset represented a subset of calls obtained from multiple recording sessions rather than all recording sessions conducted. Acoustic activities were band‐pass filtered at 0.05–1 kHz to reduce background noise and manually analysed using Avisoft‐SAS Lab Pro 5.2.13 (Avisoft Bioacoustics, Glienicke, Germany). Oscillograms were used to measure temporal features (Figure 2). Spectrograms were generated using a 512‐point fast Fourier transform (FFT), a Hann window, and 50% overlap, and were used to measure spectral features. The parameters selected to characterise the vocal repertoire were: sound duration (ms), number of pulses in a sound; pulse period (ms), measured as the time from the beginning of one pulse to the beginning of the next one; pulse duration (ms), measured as the time from the beginning of one pulse and its end; interpulse interval or IPI, measured as the time from the end of one pulse to the beginning of the next; dominant frequency (Hz), defined as the frequency with the highest energy. The acoustic data used in the analyses are provided in Table S1.

**TABLE 1:** Recording effort across study sites and dates.

Site	Date	Water temperature (°C)	Recording sessions	Minutes recorded
Anjos	07/01/25	23	6	77
Prainha	08/01/25	23	7	125
Prainha	09/01/25	23	12	133
Forno	12/02/25	22	5	72
Forno	07/12/25	23.5	3	90
Forno	08/12/25	24	2	70
Forno	11/12/25	23	4	104

*Note*: Water temperature, number of recording sessions, and total duration of video recordings (in minutes) are shown for each site and sampling date.

**FIGURE 2:**
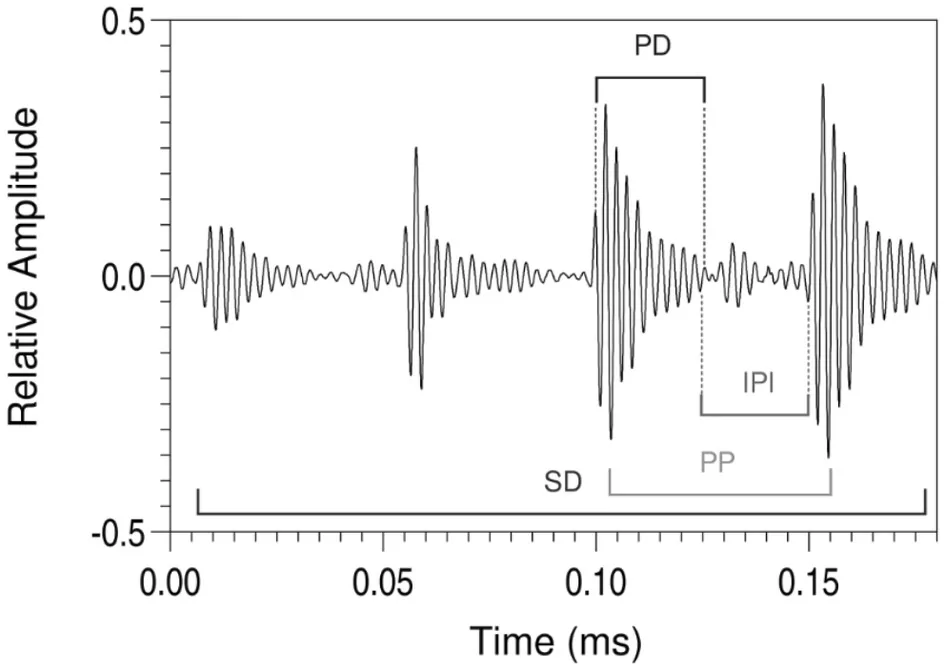
Oscillogram of a sound produced by *Stegastes fuscus* during conspecific chasing. The sound is composed of four pulses and illustrates sound duration (SD), pulse period (PP), pulse duration (PD), and interpulse interval (IPI).

### 2.4 Statistical analysis

Acoustic variables did not meet the assumptions of normality and homoscedasticity; therefore, non‐parametric tests were applied throughout. Initially, 104 sound‐associated behavioural events were classified. Agonistic behaviours (AGs) were classified according to the identity of the target individual (conspecific, heterospecific or undetermined). No significant differences were detected for any acoustic parameter except pulse duration. Because pulse duration in pomacentrids is known to be strongly influenced by individual body size (Colleye et al., 2009; Myrberg et al., 1993), rather than behavioural context, this isolated difference was not considered sufficient to justify maintaining separate agonistic categories. Consequently, these three behaviours were pooled into a single category termed AG for subsequent analyses (*n* = 77). Acoustic parameters were then compared between two behavioural contexts: agonistic and courtship (signal jump, *n* = 20). Differences between contexts were assessed using Kruskal–Wallis tests for each acoustic variable independently. Because only two behavioural contexts were compared, no post hoc pairwise tests were required.

All statistical analyses were performed in R (v.4.5.1; R Core Team, 2025) using base R functions.

## 3 RESULTS

Sound production was observed during two main behavioural contexts in *S. fuscus*: agonistic interactions and courtship‐related interactions (Figure 3), with representative oscillograms and corresponding spectrograms of the sounds produced during agonistic and courtship interactions shown in Figure 4.

**FIGURE 3:**
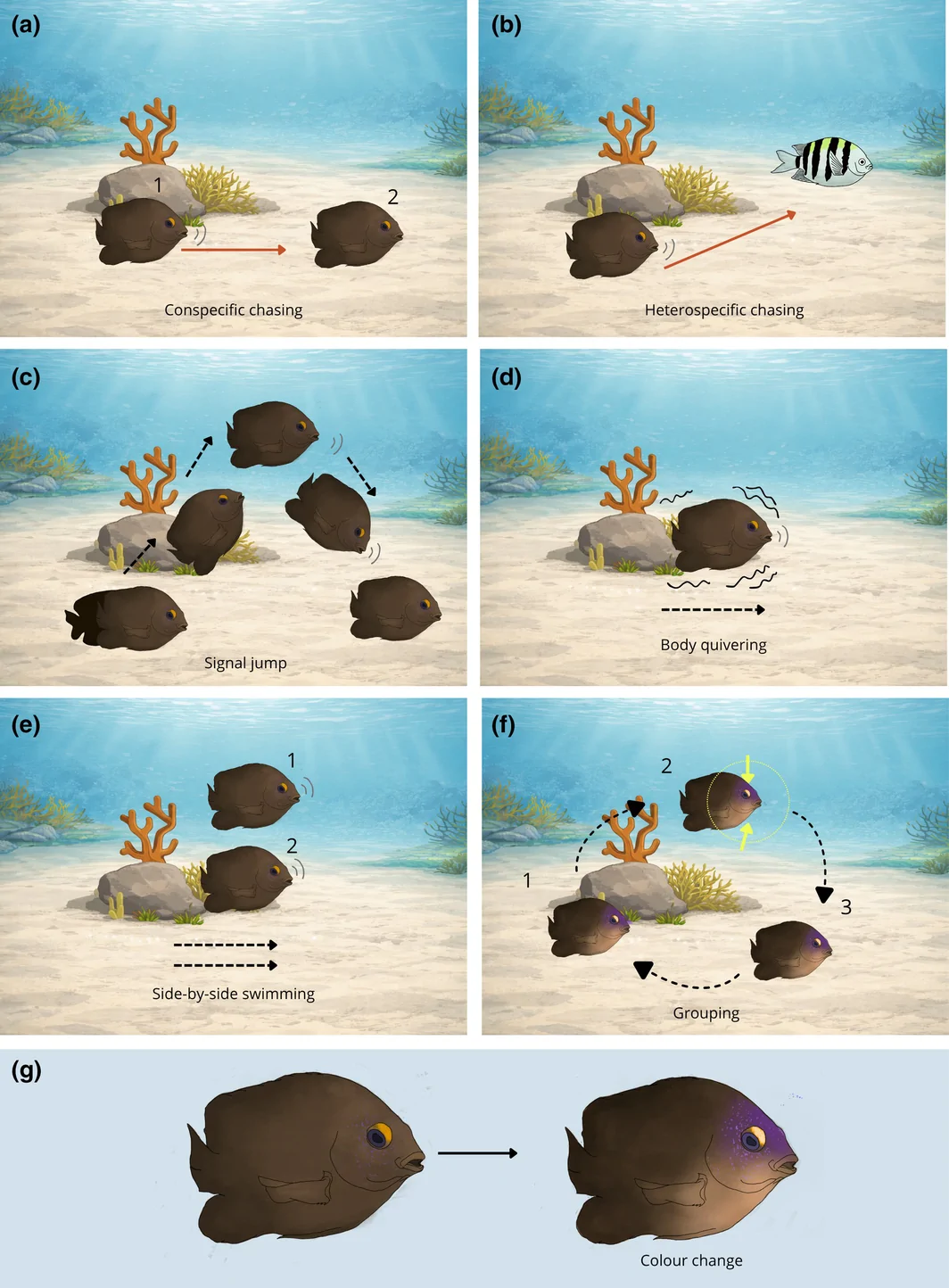
Schematic representation of behavioural patterns associated with sound production in *Stegastes fuscus* recorded in situ. (a,b) Agonistic interactions. (c–f) Courtship‐related motor patterns. Numbers above individuals identify different fish involved in each interaction. Black arrows indicate non‐agonistic movements and swimming trajectories, whereas red arrows indicate agonistic movements and pursuit direction. During grouping interactions (f), individuals frequently exhibited transient colour changes. (g) Comparison between normal and display colouration observed during courtship‐related grouping behaviour.

**FIGURE 4:**
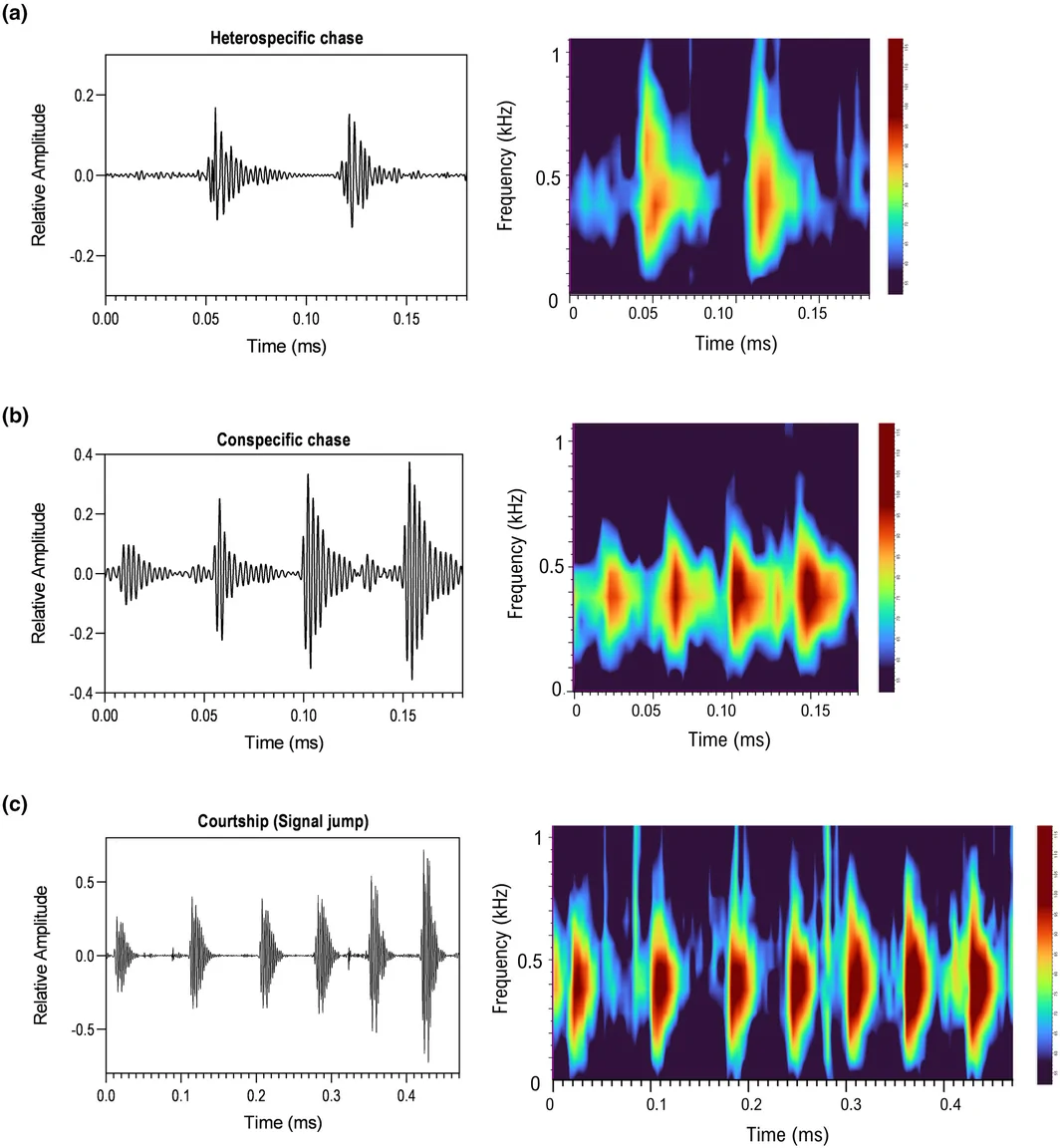
Oscillograms (left panels) and corresponding spectrograms (right panels) of sounds produced by *Stegastes fuscus* during heterospecific chasing (a), conspecific chasing (b) and courtship (signal jump) (c). Spectrograms were generated using a fast Fourier transform length of 512, Hann window and 50% overlap. Time is shown on the *x*‐axis, and relative amplitude (oscillograms) or frequency (kHz; spectrograms) on the *y*‐axis.

Agonistic interactions were subdivided into three categories based on the identity of the target individual. (I) Conspecific chase (CC), in which the calling individual chases a member of the same species (Figure 3a). (II) Heterospecific chasing (HC) involves the chase of a different species (Figure 3b). (III) Undetermined chase (UC) when the pursued individual could not be confidently identified. Reproductive interactions involved two conspecifics in proximity, the absence of overt aggression, coordinated movements, and transient body colour changes. Within this context, four behavioural patterns were identified: (IV) Signal jumps (Figure 3c) with abrupt vertical or directional darting movements; (V) Body quivering (Figure 3d) consisting of rapid, low‐amplitude body oscillations with minimal displacement; (VI) Side‐by‐side swimming (Figure 3e) during which two individuals swam parallel to one another with reciprocal movements; (VII) Grouping (Figure 3f) in which three or more individuals swam in proximity, often performing synchronised or circular movements sometimes accompanied by transient colour change. Representative examples of agonistic interactions, signal jumps, and transient colour changes are provided in Videos [Link], [Link], respectively.

Acoustic parameters differed between behavioural contexts primarily in temporal features. Courtship sounds contained more pulses and had longer durations than agonistic sounds. Significant differences were detected for number of pulses (Kruskal–Wallis test: *H* = 31.5, d.f. = 1, *p* <0.001) and sound duration (Kruskal–Wallis test: *H* = 22.8, d.f. = 1, *p* <0.001) (Figure 5, Table 2). Courtship sounds averaged 4.0 pulses per call and lasted 218.4 ± 131.2 ms, whereas agonistic sounds averaged 1.8 pulses per call and lasted 70.6 ± 73.1 ms (Figure 6). In contrast, pulse duration, pulse period, IPI, and peak frequency did not differ significantly between behavioural contexts (Kruskal–Wallis tests: *p* >0.05). Peak frequency overlapped broadly between behaviours, ranging from 280 to 560 Hz, with mean values close to 391 Hz in both contexts (Table 2). Likewise, pulse period and IPI showed substantial overlap between behaviours.

**FIGURE 5:**
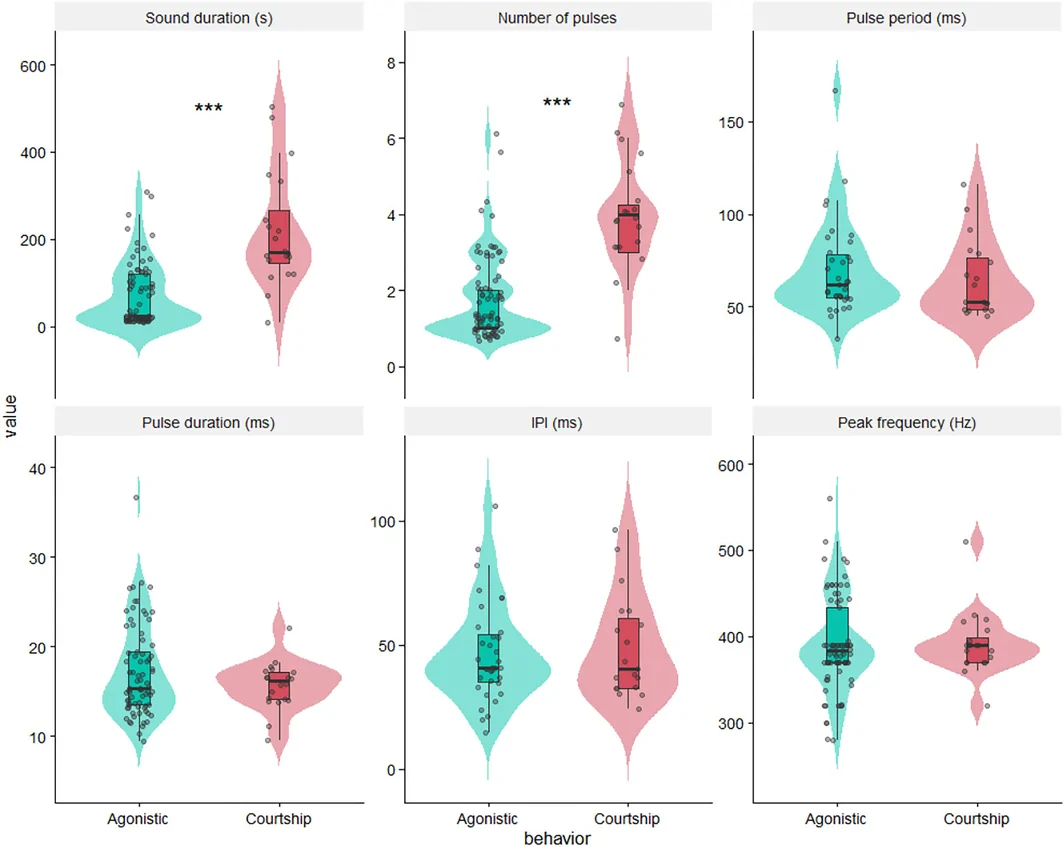
Violin plots showing the distribution of acoustic parameters of sounds produced by *Stegastes fuscus* during agonistic interactions and courtship. Acoustic variables include sound duration, number of pulses, pulse period, pulse duration, interpulse interval (IPI), and peak frequency. Boxplots indicate medians and interquartile ranges, and points represent individual observations. Asterisks denote significant differences between behavioural contexts based on Kruskal–Wallis tests (***p <0.001).

**TABLE 2:** Comparison of acoustic parameters of sounds produced by *Stegastes fuscus* during agonistic interactions and courtship (signal jump).

Acoustic parameter	Agonistic (77)	Courtship (20)
Number of pulses ***	1.8 ± 1.1	4.0 ± 1.5
Sound duration (ms) ***	70.6 ± 73.1	218.4 ± 131.2
Pulse duration (ms)	17.2 ± 5.1	15.6 ± 2.7
Pulse period (ms)	69.9 ± 25.4	64.8 ± 20.8
Interpulse interval (ms)	46.6 ± 20.1	49.0 ± 20.8
Peak frequency (Hz)	390.7 ± 53.9	391.4 ± 36.4

*Note*: Values are mean ± SD. Numbers in parentheses indicate sample size (number of sounds). Asterisks denote significant differences between behavioural contexts based on Kruskal–Wallis tests (***p <0.001).

**FIGURE 6:**
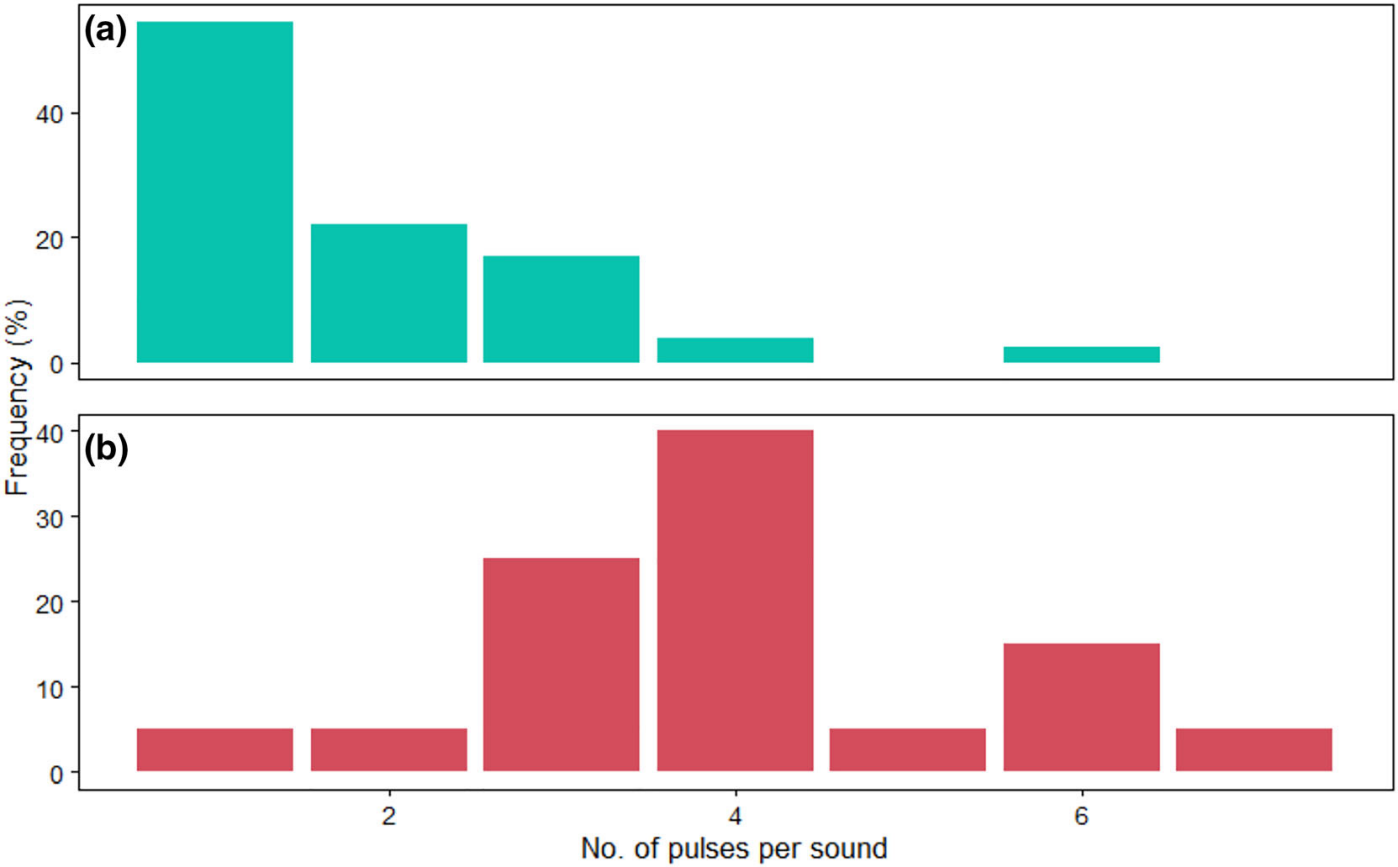
Frequency distribution (%) of the number of pulses per sound produced by *Stegastes fuscus* during (a) agonistic interactions and (b) courtship.

Sounds produced in different behavioural contexts showed a partial separation in the acoustic space defined by sound duration and number of pulses (Figure 7). Signal‐jump sounds, as associated with courtship, were characterised by longer durations due to a higher number of pulses, reflecting the positive association between call duration and pulse number. Consequently, they occupied the upper‐right region of the acoustic space. In contrast, agonistic sounds clustered at shorter durations and fewer pulses, forming a more compact distribution in the lower‐left region of the plot.

**FIGURE 7:**
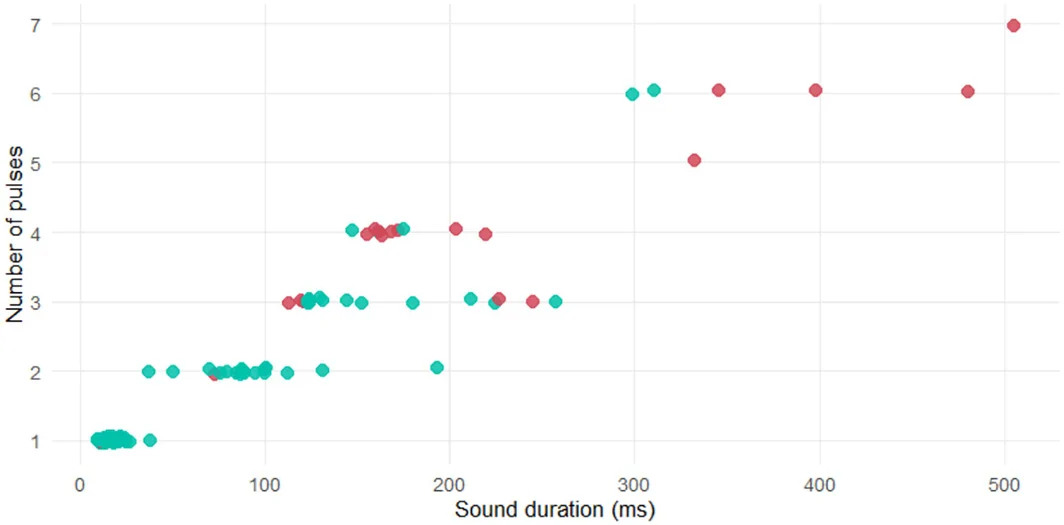
Relationship between sound duration (ms) and number of pulses in vocalisations produced by *Stegastes fuscus* during agonistic interactions (green) and courtship (pink). Each point represents an individual sound.

## 4 DISCUSSION

*S. fuscus* produced sounds associated with agonistic and courtship‐related behaviours that differed primarily in their temporal structure. Courtship calls were longer and composed of more pulses than agonistic sounds, whereas spectral features and fine pulse structure showed substantial overlap between contexts. These results indicate that behavioural context in *S. fuscus* is mainly encoded through temporal modulation of calls. However, differences in call duration and pulse number among behavioural contexts are not universal across fishes. While courtship calls were longer than agonistic ones in *S. fuscus*, the opposite pattern has been reported in species such as the cichlid *Pseudotropheus zebra* (Miguel Simões et al., 2008) and the two‐spotted goby *Pomatochistus flavescens* (Penim et al., 2024), whereas no context‐dependent differences were found in weakfish *Cynoscion regalis* (Connaughton et al., 2000). Moreover, voluntary sounds in meagre *Argyrosomus regius* were longer and contained more pulses than disturbance sounds (Pereira et al., 2020). These contrasting patterns suggest that temporal variation in fish vocalisations is species‐specific and mainly reflects the functional role of acoustic signals within each social system rather than a general rule across taxa.

The similarity in spectral properties across contexts suggests structural constraints imposed by a common sound production mechanism (Frédérich et al., 2014; Parmentier et al., 2010). In damselfishes, dominant frequency is strongly influenced by body size (Colleye et al., 2009; Maruska et al., 2007; Myrberg et al., 1993; Parmentier, 2026). Because only adult individuals of comparable body size were analysed, the similarity in peak frequency between behavioural contexts is unlikely to reflect morphological variation. Instead, behavioural differences were expressed through modifications in temporal patterning. This suggests that, as in other pomacentrids, spectral stability may be biomechanically constrained, whereas temporal structure provides greater flexibility for behavioural modulation. Temporal variation in call structure, particularly call duration and pulse number, may therefore function as a flexible signal of behavioural motivation during social interactions.

Comparative evidence suggests that the degree of acoustic diversification may vary across pomacentrid species. For example, *Dascyllus flavicaudus* and *D. albisella* exhibit a diversified acoustic repertoire with multiple distinct call types associated with different behavioural contexts (Mann & Lobel, 1998; Parmentier et al., 2010). In contrast, although *S. fuscus* displays pronounced territoriality and complex aggressive behaviours (Ferreira et al., 1998; Menegatti et al., 2003; Silveira et al., 2020), acoustic differentiation appears to occur primarily between broad behavioural categories, without evidence for consistent acoustic differentiation among agonistic subcategories. This pattern likely reflects differences in social organisation rather than differences in behavioural complexity. Adult *S. fuscus* are highly territorial, extensive farmers (Hata and Cecarelli, 2016) that defend their territories, engaging predominantly in short‐range dyadic interactions (Menegatti et al., 2003). In such a framework, modulation of temporal structure may suffice to signal motivational state during territorial defence and courtship, without requiring multiple structurally distinct call types.

Previous studies demonstrated that the identity of the intruder influences the intensity and frequency of agonistic interactions in *S. fuscus*, with higher aggression directed towards heterospecifics (Silveira et al., 2020). However, acoustic comparisons revealed no consistent differences between conspecific and heterospecific interactions in the present study, with the exception of pulse duration, a parameter known to be influenced by body size in pomacentrids. This suggests that, although opponent identity modulates behavioural investment, it does not alter the structural properties of the acoustic signal. Agonistic calls may encode motivational state or territorial defence intensity rather than opponent identity. In a visually structured benthic territorial system, discrimination between intruders is likely mediated primarily by visual cues, with acoustic signals acting as a general reinforcement of aggressive intent.

Our behavioural observations suggest that acoustic signalling in *S. fuscus* may be accompanied by visual displays, particularly during courtship‐related interactions. The combined use of acoustic and visual signals during courtship has been documented in other fishes, including damselfishes and gobies (de Jong et al., 2018; Oliver & Lobel, 2013). In *S. fuscus*, courtship‐related interactions were frequently accompanied by conspicuous colour changes and coordinated swimming displays, which sometimes occurred independently of sound emission. A similar dissociation between visual and acoustic components of courtship has been reported in other vocal fishes, where visual displays may occur without accompanying sound production (Maruska & Fernald, 2011). Such systems may rely on complementary signal components, with acoustic signals potentially reflecting behavioural motivation state while visual displays provide additional contextual cues. Colour modulation during social interactions has been reported in other damselfish species (Lobel & Kerr, 1999; Parmentier et al., 2010; Spanier, 1979), suggesting that multimodal signalling may be widespread within Pomacentridae. In *S. fuscus*, transient colour changes were most evident during grouping interactions involving three or more individuals. Because the species lacks obvious sexual dimorphism, the sex of displaying individuals could not be determined, limiting inference about potential sex‐specific roles. Although colour changes were not quantified, their consistent association with coordinated social behaviour suggests that communication in *S. fuscus* likely relies on the integration of acoustic and visual signals rather than on acoustic structure alone and motivates future studies investigating the potential role of multimodal communication in this species.

## 5 CONCLUSIONS

These findings highlight the importance of behavioural context in shaping acoustic signals and show that temporal modulation of calls may represent a flexible mechanism for signalling behavioural motivation in territorial reef fishes. Sound production in *S. fuscus* is closely linked to behavioural context and primarily modulated through changes in call organisation (e.g., number of pulses and sound duration) rather than pulse structure. Calls associated with courtship behaviours are longer and contain more pulses than agonistic sounds, irrespective of whether interactions involve conspecific or heterospecific interactions. By characterising the acoustic repertoire of a dominant territorial herbivore, this study reveals how temporal patterns structure communication in *S. fuscus* and provides a foundation for future research on sound‐mediated social interactions in functionally important reef fishes.

## AUTHOR CONTRIBUTIONS

Aléxia A. Lessa collected the data, conducted the statistical analyses, investigation, methodology, validation, visualisation, and manuscript writing, review, and editing. Viviane Barroso contributed to investigation, formal analysis, validation, and manuscript review and editing. Fábio C. Xavier contributed to supervision, investigation, formal analysis, validation, and manuscript review and editing. Carlos E. L. Ferreira contributed to supervision, funding acquisition, resources, project administration, validation, and manuscript review and editing. Eric Parmentier contributed to supervision, formal analysis, validation, and manuscript review and editing. All authors reviewed the final manuscript.

## CONFLICT OF INTEREST STATEMENT

The authors declare no conflicts of interest.

## Supporting information


**Table S1.** Acoustic and behavioural dataset used in the analyses of sound production in *Stegastes fuscus*.

**Video S1.** Representative video of an agonistic interaction in *Stegastes fuscus*.

**Video S2.** Representative video of signal‐jump behaviour in *Stegastes fuscus*.

**Video S3.** Representative video of transient colour changes during social interactions in *Stegastes fuscus*.

## Data Availability

Data are available as Supporting Information.
